# Effect of Ozone Treatment on Flavonoid Accumulation of Satsuma Mandarin (*Citrus unshiu* Marc.) during Ambient Storage

**DOI:** 10.3390/biom9120821

**Published:** 2019-12-03

**Authors:** Xiangrong Zhu, Jing Jiang, Chunxiao Yin, Gaoyang Li, Yueming Jiang, Yang Shan

**Affiliations:** 1 Hunan Key Lab of Fruits &Vegetables Storage, Processing, Quality and Safety, Hunan Agricultural Product Processing Institute, Hunan Academy of Agricultural Sciences, Changsha 410125, China; xiangrongchu@163.com (X.Z.); lgy7102@yahoo.com.cn (G.L.); 2 South China Botanical Garden, Chinese Academy of Sciences, Guangzhou 510650, China; 3 Longping branch, Graduate School of Hunan University, Changsha 410125, China; Jingjiang1995@163.com (J.J.); chunxiaoyin@scbg.ac.cn (C.Y.)

**Keywords:** satsuma mandarin, ozonation, flavonoids accumulation, antioxidant capacity, gene expression

## Abstract

This study aimed to compare the flavonoid accumulation between ozone-treated and untreated Satsuma mandarin (*Citrus* *unshiu* Marc.) fruits. The fruits exposed to gaseous ozone were found to have higher antioxidant activities and content of flavonoid during the storage period by ultra-high performance liquid chromatography (UPLC). To reveal the molecular regulation of flavonoid accumulation by ozone, chalcone synthase (*CHS*), chalcone isomerase (*CHI*), β-1,3-glucanase (*GLU*), chitinase (*CHT*), phenylalanine ammonia-lyase (*PAL*), and peroxidase (*POD*) were identified and their expression was examined by quantitative real-time polymerase chain reaction (q-PCR). These results support the promising application of ozone treatment as a safe food preservation technique for controlling postharvest disease and extending shelf-life of harvested Satsuma mandarin.

## 1. Introduction

Citrus fruit, the most important fruits produced in commercial scale in the world and China, are susceptible to postharvest fungal decay by *Penicillium italicum* and *Penicillium digitatum,* resulting in important economic losses [1]. The use of chemical fungicides to control fungal diseases during the postharvest storage of citrus fruits may bring potential risks to human health and the environment [2]. Therefore, there is an obvious demand for other approaches to reduce decay and to prolong postharvest life of citrus fruits [3].

Ozone (O_3_) was approved by the U.S. Food and Drug Administration (FDA) as an anti-microbial agent for food storage in 2001 [4]. Ozone readily decomposes into O_2_ without leaving any residue and accumulation of toxic secondary metabolites [5]. Ozone treatment has been effective in prolonging the shelf-life of papaya [6], wine grapes [7], black mulberries [8], peppers [9], strawberry [10], cucumbers and zucchini [11]. Previous research showed that O_3_ treatment increases antioxidant capacity, total phenol, and flavonoid content in table grapes [12] and pears [13]. Furthermore, it was noted that the strong oxidative properties of O_3_ could influence antioxidant and defense-related enzyme activities [14,15,16]. Total phenolics content has previously been found to be increased in ozone-treated banana [17], papaya [18], kiwi [19], green asparagus [20], and globe artichoke [21] when compared with control fruits and vegetables. The possible mechanism may be related to increased activity of phenylalanine ammonia-lyase (PAL) [22,23], SOD [16,24] and peroxidase (POD) [25] or decreased activity of polyphenol oxidase (PPO) [20,26]. These enzymes are capable of degrading polymers of fungal cell walls and are therefore thought to be involved in plant defence mechanisms by reducing fungal growth, and thus, lowering the rate of decay [16,23].

However, information available on changes of flavonoid content, antioxidant capacities as well as related-gene expression in Satsuma mandarin during ozone treatment is limited. Therefore, the objective of this study was to investigate the effects of ozone treatment on content of flavonoid, the antioxidant capacity, key genes expression, and some important characteristics, such as respiratory rate, decay rate, and colour change of Satsuma mandarin and to fully understand the inter-relationship among flavonoid compounds, the antioxidant capacity, key genes, as well as the association with postharvest quality.

## 2. Materials and Methods

### 2.1. Fruit Material

Satsuma mandarin (*Citrus unshiu* Marc.) fruit was harvested from a large-scale commercial orchard in Shaoguan city, Guangdong Province, China. They were transported to the laboratory, selected for uniformity of shape, size, color, appearance, and then randomly divided into six groups stored at ambient temperature (25 ± 1 °C). The citrus peels were manually separated and frozen in liquid nitrogen immediately and kept at −80 °C until needed for flavonoid and q-PCR analysis.

### 2.2. Ozone Treatments

Ozone was pumped into the container from ozone generator (Tianjin, China), and the concentration was kept at 2.5 μg L^−1^ for 24 h. Quality analyses of different samples were carried out over 30 consecutive days, during 1 d, 4 d, and 30 d of storage. Thirty fruit samples were taken from a closed container at each sampling date.

### 2.3. Colour Measurement

The Minolta CR-400 Chroma Meter (Minolta Corp., Osaka, Japan) was used to obtain fruit skin colour from three different locations around the equator of the fruit. The colour was recorded using a* and b* scale, where a* represents the colour between green (-a*), and red (+a*) and b* represents the colour between blue (-b*) and yellow (+b*). The a* and b* values were converted into Hue angle [H = tan^−1^ (b*/a*)]. Three replicates at each period were performed for each analysis.

### 2.4. Respiratory Rate and Decay Rate

Respiration rate of citrus fruit was determined, referring to the method of Huang and Jiang [27]. Four whole citrus fruits were weighed and sealed into a plastic box that connected the CO_2_/H_2_O Analyzer (LI-6262, LI COR, USA) before the amount of CO_2_ was recorded for 5 min. Respiration rates were expressed as the rate of CO_2_ production as calculated on a fresh weight basis (mg × kg^−1^ × s^−1^).

During the course of the experiment, the number of fruits showing decay symptoms was visually estimated. Fruits with visible mould growth were considered to be rotted, and the percentage of decayed fruits was expressed as the decay rate.

### 2.5. Sample Preparation

Sample powder of fruit peel was extracted with 10 mL of 70% methanol for 30 min by ultrasonic extraction. The extracting solution was subsequently centrifuged at 3000× *g* for 10 min, and the residue was extracted again in the same way. It was then passed through a 0.2 μm membrane filter and stored at 4 °C before analysis.

### 2.6. Antioxidant Activity Assay

The antioxidant activity was evaluated by DPPH (2,2-diphenyl-1-picrylhydrazyl) and ABTS (2,2′-azino-bis(3-ethylbenzthiazoline-6-sulphoic acid) methods. The scavenging activity on DPPH radical was determined according to the method of Yang et al. [28] with some modifications. 0.4 mL of the diluted sample extracting solution was mixed with 3.5 mL of 0.12 mM DPPH solution in methanol. The absorbance was measured at 517 nm. The ABTS assay was measured according to the method established in the literature [29]. The ABTS^·+^ working solution was prepared by diluting the stock solution accurately with sodium acetate buffer to obtain an absorbance of 0.70 ± 0.01 at 734 nm. 0.4 mL of the diluted samples were added to 3.6 mL of the ABTS**^+^** working solution, having reacted for 30 min at room temperature. The results were calculated as mmol Trolox TE × kg^−1^ dry weight.

### 2.7. Instrumentation and Chromatographic Conditions

Acquity ultra-performance liquid chromatography (UPLC)^®^ H-Class (Waters, MA, USA) and a BEH C18 column (1.7 µm, 2.1 mm × 50 mm) were used to separate and quantitate the flavonoid in citrus peel samples. The binary mobile phase consisted of 0.5% aqueous formic acid (A) and HPLC grade methanol (B). A gradient elution at a flow rate of 0.3 mL min^−1^ was employed as follows: 5% B at 0–7 min, 5–12% B at 7–8 min, 12–17% B at 8–12 min, 17% B at 12–17 min, 17–30% B at 17–18 min, 30% B at 18–26 min, 30–70% B at 26–42 min and 70–5% B at 42–45 min. Eleven flavonoid standard references were purchased from Chengdu Must Bio-Technology Corp. (Chengdu, China, purity >98%).

### 2.8. q-PCR Analysis

Total RNA was isolated from 5 g of frozen skin tissue powder using TriZol Reagent (Invitrogen, USA) and then treated with DNase I at 37 °C for 1 h to eliminate genomic DNA contamination. The first-strand cDNA was synthesized from 1 µg of total RNA using a PrimeScript^®^RT reagent kit with gDNA eraser (TAKARA-RR036A, Dalian, China). The cDNA was diluted 10-fold, and 2 µL of the diluted cDNA was used as the template for q-PCR analysis.

The quantitative real-time PCR (q-PCR) experiment was performed on the Step One Plus™ (Applied Biosystems, Foster City, CA, USA). Fluorescent intensity data were analysed by 7500 Fast System Software 2.0.1. The q-PCR assay was performed under the following parameters: 95 °C for 5 min, 40 cycles at 95 °C for 5 s, 60 °C for 5 s, and 72 °C for 34 s. The 2^−ΔΔCT^ method was used to calculate the transcript values relative to the endogenous actin gene.

Chalcone synthase (*CHS*) and chalcone isomerase (*CHI*) gene-specific primers were designed on the sequences reported by Wang et al. [30]. Chitinase (*CHT*), phenylalanine ammonia-lyase (*PAL*), and β-1,3-glucanase (*GLU*) gene-specific primers were designed on the sequences reported by Lu et al. [31].

### 2.9. Statistical Analysis

All data analyzed were presented as the mean ± standard deviation (SD) and were subjected to analysis of variance (ANOVA). To compare the mean differences, the effect of ozone treatment and duration on fruit quality (respiratory, fruit colour, and decay) and the content of flavonoid in fruit peel extract and their antioxidant capacity were evaluated by Duncan’s multiple range tests (*p* < 0.05). Differences resulting in *p*-values of less than 0.05 were considered to be statistically significant.

## 3. Results and Discussion

### 3.1. Physical and Physiological Analyses

During postharvest storage (Table 1), the respiration rate of ozone treatment was lower than the control check (CK). The respiration rate of the 4 d fruit was similar to that of 30 d fruit following ozone-treated fruit. The results indicate that the ozone treatment can reduce the respiration rate at the earlier storage stage and inhibit the increased respiratory rate of fruit at the later storage stage, and thus reduce the nutrition loss in fruit.

Hue index is the most commonly used parameter to indicate the colour changes of citrus fruit in storage. Table 1 shows CIELab color parameters of postharvest citrus fruits at the end of the storage process. The Hue angle value of fruit treated by ozone was significantly higher (less yellow) than those untreated. Changes in hue resulting from ozone treatment became very small by 30 days. The results demonstrated that lower maturity and degradation of chlorophyll and better keeping green effects were obtained with ozone treatment.

By statistical analysis (Table 1), the rotting rate after treatment were 14.0% during the storage of 30 d, the rotting rate lower than the CK of 34.7%. The results indicated that ozone treatment could decrease the rate of fruit rot.

### 3.2. Effects of Ozone on the Flavonoids Content of Citrus Peel

**Figure 1:**
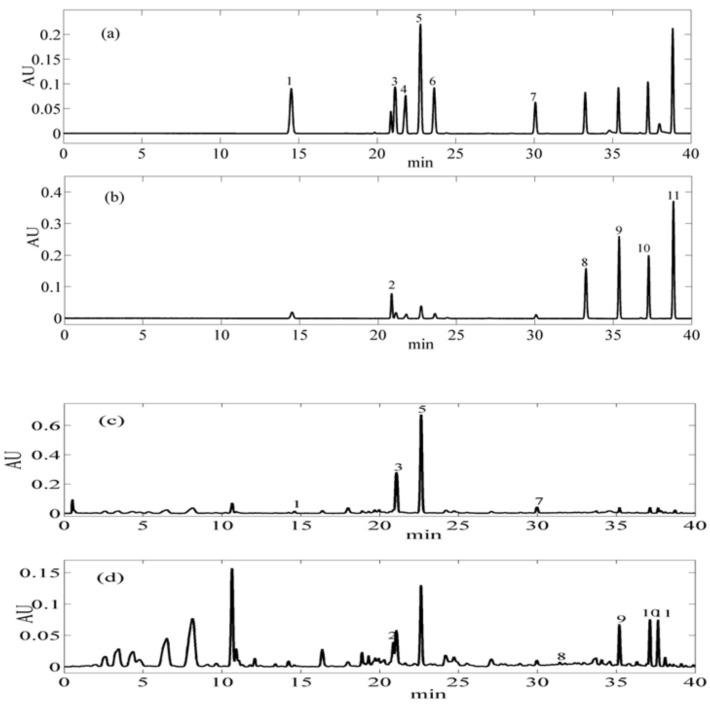
Ultra-high performance liquid chromatography (UPLC) chromatograms of eleven flavonoid mixed standards and citrus mandarin peel samples at 280 nm and 330 nm. (**a**) The mixed standard of naringin, narirutin, hesperidin, neohesperidin, taxifolin, didymin at 280 nm; (**b**) The mixed standard of rutin, diosmetin, sinensetin, nobiletin, and tangeretin at 330 nm; (**c**) Sample of narirutin, hesperidin, taxifolin, didymin at 280 nm; (**d**) Sample of rutin, diosmetin, sinensetin, nobiletin, and tangeretin at 330 nm.

The content of flavonoid in the samples was determined by the established method [32]. The chromatogram is shown in Figure 1; good separation of the flavonoid can be achieved in mixed standards and samples.

The effects of ozone treatment on the flavonoid content of mandarin citrus peel are shown in Table 2. In the peel of mandarin citrus, the content of nine flavonoid compounds except naringin and neohesperidin were detected and analysed. During the storage and ozone treatment period, the content of narirutin, hesperidin, and rutin displayed an increasing trend. Hesperidin and narirutin were found to be the major compounds. Similar results for mandarin citrus by UV radiation have been reported by Shen et al. [32]. In our research, upon exposure to ozone for 1 day, the total flavonoid content of the samples increased significantly (*p* < 0.05) by 5.8%. The flavonoid content of 4 d and 30 d increased significantly by 10.8% and 11.3%, respectively.

Remarkably, the ozone treatment leads to the result that the flavonoid accumulation patterns of different branches changed significantly. During 4 d and 30 d, narirutin content was increased by 27.3% and 29.9%, respectively, as compared to the control, and for hesperidin, the increment caused by the ozone treatment were 2.88% and 5.75%, respectively. For the content of four main flavanone glycosides (FGs) including narirutin, hesperidin, taxifolin and didymin on 4 d, the control fruits were 6527.7 ± 32.6, 15644.2 ± 263.8, 135.2 ± 2.2 and 1255.7 ± 1.7 mg × kg^−1^, while the ozone-treated fruits were 8306.2 ± 102.4, 16095.1 ± 81.1, 149.9 ± 4.7 and 1635.3 ± 2.3 mg × kg^−1^, indicating a general enhanced tendency of FGs in Satsuma mandarin fruits treated with ozone. The levels of the three determined polymethoxylated flavones (PMFs, sinensentin, nobiletin, and tangeretin) declined significantly in the ozone-treated fruits. The content of sinensentin, nobiletin, and tangeretin were 478.6 ± 20.1, 759.9 ± 13.9 and 313.8 ± 4.3 mg × kg^−1^ in ozone treated fruits on 30 d, while they were 398.4 ± 5.1, 653.8 ± 1.5 and 307.1 ± 3.4 mg × kg^−1^ for CK on 30 d, respectively. For rutin, which was the only determined flavonol glycoside, the increment caused by the ozone treatment on 4 d and 30 d were 5.9% and 22.8%, respectively. For diosmetin, as the only flavone, the content first rose and then took a downward trend afterward.

### 3.3. Analysis of Antioxidant Capacity

The antioxidant activity of plant extracts was not be evaluated by using only one method due to the complexity of chemical composition and oxidative stress [33]. Therefore, DPPH and ABTS methods were used to evaluate the antioxidant activity in this study (Table 3).

The antioxidant activity of citrus fruit peel, as estimated by the DPPH assay, increased with ozone treatment. Upon exposure to ozone on 1 d and 4 d, the antioxidant activity of citrus peel extract increased (*p* < 0.05) by 8.7% and 8.5%, respectively. While on 30 d of storage, the DPPH assays of ozone-treated fruit peel only showed a slight increase of 3.1%. On the other hand, when the antioxidant activity of citrus fruit peel was determined with the ABTS method, compared to control fruit, a slight increase of 4.2%, 2.1%, and 1.5% were measured on 1 d, 4 d, and 30 d, respectively. The trend obtained from both DPPH and ABTS assays was similar to that of total flavonoids, so it can be deduced that flavonoids compounds are major antioxidants in citrus peel.

### 3.4. Gene Expression Involved in Flavonoid Synthesis

**Figure 2:**
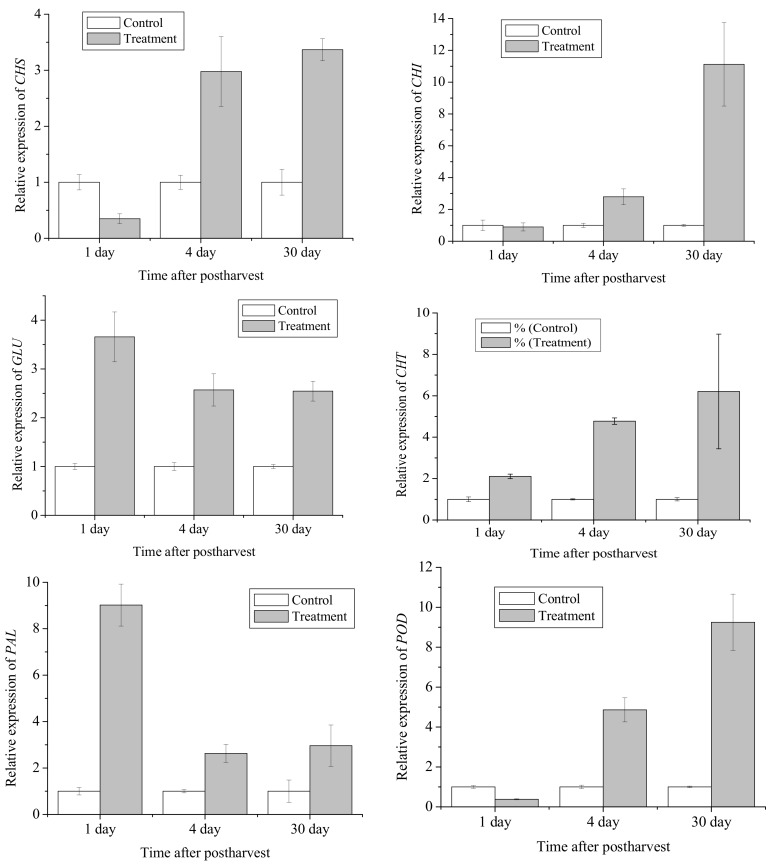
Expression patterns of six genes determined by q-PCR in the skin of citrus mandarin peel. Each data point represents a mean ± standard error (n = 3).

To understand the molecular mechanism of the differences observed in antioxidant activity changes and flavonoid accumulation following ozone fumigation, expression of *CHS*, *CHI*, *GLU*, *PAL, CHT,* and *POD* were analysed by q-PCR. The results are shown in Figure 2. The q-PCR analysis was carried out on citrus peel samples collected on 1 d, 4 d and 30 d, due to the correspondence between ozone fumigation and antioxidant activity and flavonoid content at those time points.

Significant upregulations in *CHS* and *CHI* levels were observed in the treated citrus peel. The mRNA levels of *CHS* were increased by 3.0- and 3.4-fold in ozone-treated on 4 d and 30 d. The levels of *CHI* transcripts in ozone treated citrus peel showed an increment on day 4, with the highest induction of an 11.1-fold increase observed on 30 d. The expression of the *GLU* gene in the treated peel samples reached the highest level after 1 d storage and then declined on 4 d and 30 d. However, the overall gene expression levels in samples obtained from ozone treatment were significantly higher than the levels observed in the control citrus fruit peel. The treatment of Satsuma mandarin with ozone maintained the increases in *CHT* and *PAL* mRNA transcripts during different storage periods. The expressions of *CHT* were 2.1, 4.8, and 6.2-fold, and the expressions of *PAL* were 9.0-, 2.6-, and 3.0-fold higher than in the controls on 1 d, 4 d, and 30 d, respectively.

### 3.5. Gene Expression of POD

The expression of *POD* in the citrus peel was affected significantly by the ozone treatment. Although the *POD* transcripts in the ozone-treated mandarin peel decreased on the first day after postharvest, the treatment of Satsuma mandarin with ozone-induced *POD* transcription, as shown by an approximately 4.9-fold increase on 4 d and was 9.2-fold higher on 30 d than the control, respectively. The expression of *POD* was rapidly increased in ozone treatment during storage, and the higher expression of *POD* in the ozone-treated citrus fruit would favour protecting against the cell membrane damage.

## 4. Discussion

The respiration rate of citrus fruit is an important index reflecting the effect of metabolism after storage. Our study indicated that ozonation could inhibit the respiration of postharvest Satsuma mandarin, and the reason is that ozone triggers the formation of reactive oxygen species (ROS), which lead to stomata closure [34]. Similar results were obtained by other investigators. Zhang et al. [35] showed that ozonated water treatment caused much reduction in respiration rates of fresh-cut celery. Chauhan et al. [36] found that respiration rates of carrot sticks washed by ozonated water were significantly lower than that of non-treated after 30 d of controlled atmosphere storage.

Colour changes have been used to assess postharvest fruit senescence by ozone treatment. Zambre et al. [37] observed that the development of red colour was delayed during shelf-life storage of tomatoes treated by gaseous ozone. Sandhu et al. [38] reported that ozone could react with carotenoids consisting of the multiple conjugated double bonds in wheat flour, thereby decreasing b*-value (yellowness). Bermudez-Aguirre and Barbosa-Canovas [39] treated carrots with ozone gas at concentrations between 10 and 115 mg × L^−1^, the lightness value (L*) were significantly increased, which suggests that the orange-red peel color was bleached by the ozone treatment. Our results showed that treatment with ozone could maintain the original quality of the citrus fruit regarding its color characteristics.

Based on the effect of ozone treatment on quality and physiological parameters, our study demonstrated that the antioxidant activity of Satsuma mandarin peel increased as fruit storage time increased and was further enhanced by exposure to ozone from 1 d to 30 d. The antioxidant capacity increased when total flavonoids (TF) content increased, and this was expected because there was a highly significant correlation between antioxidant capacity and TF. The correlation coefficients between DPPH/ABTS and TF were 0.93 and 0.86, respectively. Yeoh and his colleagues [18] obtained similar results in a study of the antioxidant activity measured by DPPH and ferric reducing ability of plasma (FRAP) assays of fresh-cut papaya. Antioxidant activity increased significantly (*p* < 0.05) after exposure to 9.2 µl × L^−1^ of ozone.

Flavonoids have been shown to be the major contributors to the scavenging of oxygen-derived free radicals in plant-derived food [40,41]. Studied have reported a positive correlation between the antioxidant capacity and flavonoid content [42,43]. The DPPH and ABTS free radical scavenging activity of fruit directly correlated with the prevention of fruit senescence and quality during storage [44,45]. Certain bioactive compounds, i.e., flavonoid concentrations, are the primary reason for the higher DPPH and ABTS scavenging capacity of fruit [46]. An increase in antioxidant activity after continuous exposure to gaseous ozone may be due to the activation of several intracellular enzymes that are responsible for the control of endogenous antioxidant defence in harvested fruits and vegetables [47].

Flavonoids are able to act as antioxidants, and their antioxidant potential is determined by their chemical structure [48]. Mandarin is rich in hesperidin, followed by narirutin [49,50]. Hesperidin shows high antioxidant activities, while that of narirutin is about ten times less efficient [51]. Indeed, the antioxidant activity of hesperidin is explained by the presence of methoxy (-OCH_3_) and hydroxyl (-OH) groups in the B ring. These two kinds of organic functional groups have free radical scavenging and antioxidant activity comparable to the one observed due to the existence of a catechol group. In contrast, narirutin contains only one hydroxyl group in the B ring. Therefore, antioxidant activity is much lower [52]. Our UPLC analysis showed that the flavonoid composition of mandarin peel following ozone treatment is characterized by the presence of high-level hesperidin at the end of storage.

The expression of *CHS* and *CHI* genes was significantly correlated to flavonoid accumulation in the fruit peels during postharvest storage and ozone treatment of Satsuma mandarin (r = 0.99 for *CHS* and 0.78 for *CHI*). It was consistent with previous studies examining UV-B treatment of peach [53] and fruit development using Rio Red grapefruit peels [54]. Meanwhile, expression of both genes was correlated with the content of narirutin and hesperidin, and the correlation coefficients between gene expression and accumulation of the above two dominant flavonoids had a range of 0.73–0.99. A similar result was also observed in Wang’s study [30]: total flavonoid, narirutin, and hesperidin content, as well as expression of *CHS* and *CHI* genes, decreased gradually in the peels of Satsuma mandarin during fruit maturation.

*GLU* and *CHT* have the ability to directly hydrolyse fungal cell walls in plant pathogens, thereby releasing *β*-1, 3-glucan, and chitin, which then trigger host defence responses [31]. *PAL* is involved in the biosynthesis of flavonoids of citrus fruit and could be stimulated by various abiotic stresses such as wounding, infection, and UV-C exposure [53]. In the current study, *GLU*, *CHT* and *PAL* transcript expression increased in Satsuma mandarin peels treated with ozone during storage, and these values were significantly higher than that in control fruits. Moreover, the level of total flavonoids increased and was significantly higher in ozone-treated fruits when compared to control after 4 d and 30 d of storage. It is proposed that the inductive effect of ozone on the defense-related flavonoid compounds in Satsuma mandarin contributed to the observed resistance to fruit decay symptoms when they started to appear. Similar biostimulant activities by ozone have also been reported in other horticultural crops. Ozone elicits the accumulation of pathogenesis-related (PR) proteins *GLU* and *POD* in kiwi fruit postharvest ripening [55,56]. Ozone also induced and elevated the gene expression of *GLU, CHT* and *PAL* in ‘Redglobe’ and ‘Sugraone’ table grapes [57], and thus, inhibited the development of postharvest fungal decay.

Increased *POD* activity was related to enhanced plant disease resistance [58]. In addition, *POD* was related to the accumulation of flavonoid and phenolic compounds. The results showed that ozone treatment induced higher gene expression levels of *POD* during the latter storage period, corresponding to the total flavonoid content which was significantly higher than that of the control over the same period, suggesting that in harvested Satsuma mandarin fruits, the increased expression of *POD* gene by ozone treatment was correlated to the levels of total flavonoids. Similarly, Sachadyn-Król et al. [59] found that the *POD* activities were higher in pepper fruit treated with gaseous ozone when compared to that in control samples. The activities of *POD* and *PAL* were higher in ozone fumigated papaya fruit than in untreated fruit after 14 days of storage [60]. These results also suggest that ozone treatment enhanced expression *of POD,* and this contributed to the activation of the antioxidant defence system in Satsuma mandarin fruits.

## 5. Conclusions

Continuous exposure of Satsuma mandarin fruits to ozone resulted in retarded respiration rate, reduced rot rate, and better appearance. The results indicate that fumigation with the ozone-induced changes in postharvest Satsuma mandarin is an effective strategy to regulate the concentration of flavonoid compounds, and it is able to induce changes in gene expression level and increase both DPPH and ABTS values. The application of ozone seems to be a feasible solution to reduce quality loss during the storage of Satsuma mandarin.

## Figures and Tables

**Table 1:** The respiration rate, hue angle value (Hue^0^) and decay rate of mandarin citrus fruit. Each value is presented as a mean ± standard deviation (n = 3). Values in the rows denoted with different superscript letters (a–f) indicate significant differences at *p* < 0.05.

	CK-1 d	O_3_-1 d	CK-4 d	O_3_-4 d	CK-30 d	O_3_-30 d
Respiratory rate (mg × kg^−1^ × s^−1^)	16.2 ^a^ ± 1.5	12.2 ^b^ ± 0.8	10.2 ^c^ ± 1.10	8.4 ^d^ ± 0.7	15.7 ^a^ ± 4.89	8.2 ^d^ ± 3.9
Hue^0^ Decay rate (%)	98.3 ^a^ ± 3.3 -	104.7 ^b^ ± 4.0 -	87.2 ^c^ ± 2.9 -	100.7 ^d^ ± 5.4 -	76.0 ^e^ ± 2.7 34.7 ^a^	79.2 ^f^ ± 3.9 14.0 ^b^

**Table 2:** The flavonoid content (mg × kg^−1^ dry weight) in the peel of mandarin citrus. Each value is presented as a mean ± standard deviation (n = 3). Values in the rows denoted with different superscript letters (a–f) indicate significant differences at *p* < 0.05.

	CK-1d	O_3_-1d	CK-4d	O_3_-4d	CK-30d	O_3_-30d
Naringin	-	-	-	-	-	-
Narirutin	7661.8 ^a^ ± 79.5	6599.2 ^bc^ ± 102.9	6527.7 ^b^ ± 32.6	8306.2 ^d^ ± 102.4	6689.1 ^c^ ± 34.2	8686.6 ^e^ ± 92.3
Hesperidin	13,136.6 ^a^ ± 144.0	14,851.7 ^b^ ± 275.2	15,644.2 ^c^ ± 263.8	16,095.1 ^d^ ± 81.1	15,623.4 ^c^ ± 59.5	16,522.2 ^e^ ± 159.8
Neohesperidin	-	-	-	-	-	-
Taxifolin	119.7 ^a^ ± 0.8	136.6 ^b^ ± 0.3	135.2 ^b^ ± 2.2	149.9 ^c^ ± 4.7	145.6 ^c^ ± 5.2	136.5 ^b^ ± 2.3
Didymin	1355.8 ^a^ ± 0.6	1311.4 ^a^ ± 4.3	1255.7 ^b^ ± 1.7	1635.3 ^c^ ± 2.3	1584.9 ^d^ ± 86.6	1521.5 ^e^ ± 13.5
Rutin	1765.8 ^a^ ± 39.5	2583.8 ^bd^ ± 83.8	2473.5 ^c^ ± 86.8	2619.6 ^d^ ± 46.5	2499.4 ^bc^ ± 24.8	3068.7 ^e^ ± 15.3
Diosmetin	67.5 ^a^ ± 1.8	86.8 ^b^ ± 2.3	81.1 ^c^ ± 0.1	101.0 ^d^ ± 0.1	100.0 ^d^ ± 3.1	71.5 ^e^ ± 1.0
Sinensetin	464.6 ^ab^ ± 20.6	469.8 ^abc^ ± 9.1	446.7 ^a^ ± 15.3	494.8 ^c^ ±11.8	478.6 ^bc^ ± 20.1	398.4 ^d^ ± 2.9
Nobiletin	626.9 ^a^ ± 8.0	645.6 ^ac^ ± 2.2	628.6 ^ac^ ± 6.3	728.2 ^b^ ± 9.8	759.9 ^b^ ± 13.9	653.8 ^c^ ± 1.5
Tangeretin	311.4 ^a^ ± 7.2	294.9 ^b^ ± 2.9	292.2 ^b^ ± 1.0	309.6 ^a^ ± 2.1	313.8 ^a^ ± 4.3	307.1 ^a^ ± 3.4
Total	25,510.1 ^a^ ± 302.0	26,979.8 ^b^ ± 483.0	27,484.9 ^c^ ± 409.8	30,439.7 ^d^ ± 260.8	28,194.7 ^e^ ± 251.7	31,366.3 ^f^ ± 292.0

**Table 3:** The Results of 2,2-diphenyl-1-picrylhydrazyl (DPPH) and 2,2′-azino-bis(3-ethylbenzthiazoline-6-sulphoic acid (ABTS) free radical scavenging activity (mmol Trolox TE × kg^−1^ dry weight) in the peel of mandarin citrus. Each value is presented as a mean ± standard deviation (n = 3). Values in the rows denoted with different superscript letters (a–d) indicate significant differences at *p* < 0.05.

	CK-1d	O_3_-1d	CK-4d	O_3_-4d	CK-30d	O_3_-30d
DPPH	44.0 ^a^ ± 0.2	47.6 ^b^ ± 0.2	47.4 ^b^ ± 0.1	51.4 ^c^ ± 0.3	50.9 ^c^ ± 0.8	52.7 ^d^ ± 0.2
ABTS	113.3 ^a^ ± 1.3	118.0 ^b^ ± 0.4	119.0 ^b^ ± 0.4	121.9 ^c^ ± 0.4	124.1 ^d^ ± 1.8	125.4 ^d^ ± 0.1
